# Primary Leiomyosarcoma of the Mesentery: A Case Report With Review of Literature

**DOI:** 10.7759/cureus.10777

**Published:** 2020-10-03

**Authors:** Saif Affas, Mohamad F Ayas, Juliann M Mendes, Tarik Hadid

**Affiliations:** 1 Internal Medicine, Ascension St. John Hospital and Medical Center, Detroit, USA; 2 Pathology and Laboratory Medicine, Ascension St. John Hospital and Medical Center, Detroit, USA; 3 Oncology, Ascension St. John Hospital and Medical Center, Detroit, USA

**Keywords:** primary leiomyosarcoma, leiomyosarcoma, mesentery, gist, literature review of disease

## Abstract

Mesenteric leiomyosarcoma (LMS) is a rare gastrointestinal mesenchymal tumor. It was often misdiagnosed as a gastrointestinal stromal tumor (GIST) until the introduction of immunohistochemistry staining (IHC) in 1998. Currently, a positive IHC staining for smooth muscle markers represents the main diagnostic modality. Herein, we present a case of Mesenteric LMS in a 68-year-old woman, who presented with nausea, vomiting, and abdominal pain and was found to have a right-sided mesenteric tumor encasing the right ureter, with right hydroureteronephrosis. The patient underwent surgical removal of the tumor and IHC stains were compatible with LMS. She achieved remission until she developed recurrence 12 months after initial diagnosis and subsequently expired due to postoperative complications when re-resection was attempted. Typically, mesenteric LMS carries a poor prognosis with a propensity for hematogenous metastasis. In the absence of a standardized protocol for therapy, early surgical resection is the only known curative modality but with a high risk of recurrence.

## Introduction and background

Leiomyosarcoma (LMS) is a rare malignant soft tissue tumor that originates from smooth muscle cells, with an incidence rate of 1 per 350,000 cases [1,2]. Although mainly found in the retroperitoneum, uterus, and medium to large abdominal vessels, it can rarely arise from the mesenteric smooth muscles [3,4]. Prior to the development of immunohistochemical (IHC) staining, LMS was frequently misdiagnosed as a gastrointestinal stromal tumor (GIST), given the histological similarities as both entities are under the umbrella of gastrointestinal mesenchymal tumors. IHC is currently considered essential for the accurate diagnosis of LMS [1,5,6]. Similar to most soft tissue sarcomas, LMS tends to grow along the tissue planes and thus can compress surrounding structures, leading to the formation of a pseudo-capsule with fingerlike projections that can infiltrate adjacent tissues [7].

Case report

A 68-year-old woman with a history of type 2 diabetes mellitus, essential hypertension, and latent tuberculosis presented to the hospital with generalized fatigue, weakness, and unintentional 10-pound weight loss over two months. She also reported abdominal pain, nausea, vomiting, and diarrhea for two days but denied fever, chills, and night sweats. At presentation, vital signs were normal. Her examination showed a soft, nondistended abdomen with mild right upper quadrant tenderness on palpation, but without hepatosplenomegaly, and with a negative Murphy’s sign.

Laboratory studies showed a hemoglobin of 9.9 gm/dl, and a white blood cell of 9.7 Thou/cu mm. Blood smear showed normochromic normocytic anemia with mild anisocytosis. The iron panel was normal apart from mildly elevated serum ferritin with a normal vitamin B12 and RBC folate level. The patient had a colonoscopy done one month before her presentation in another facility which was reportedly negative.

**Figure 1:**
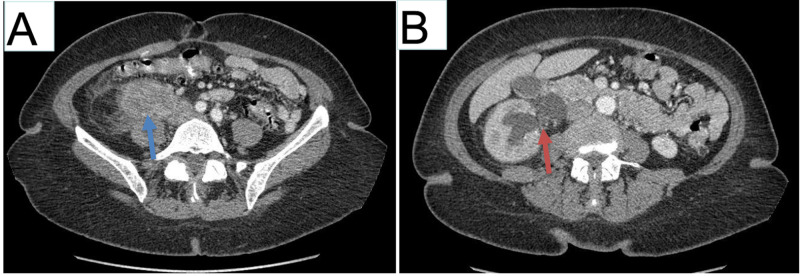
CT findings (A) Solid and cystic mass in the right retroperitoneum, measured 5.7 x 4.2 x 5.7cm^3^ in size (blue arrow). (B) Right hydronephrosis and proximal and mid-right hydroureter (red arrow) due to external compression effect of the mass.

An enhanced computed tomography (CT) of the chest, abdomen, and pelvis revealed a 5.7 x 4.2 x 5.7 cm^3^ right-sided mesenteric mass causing extrinsic compression of the right mid-ureter with prominent right-sided hydroureteronephrosis (Figure 1). No pulmonary, hepatic lesions, or lymphadenopathy were identified.

**Figure 2:**
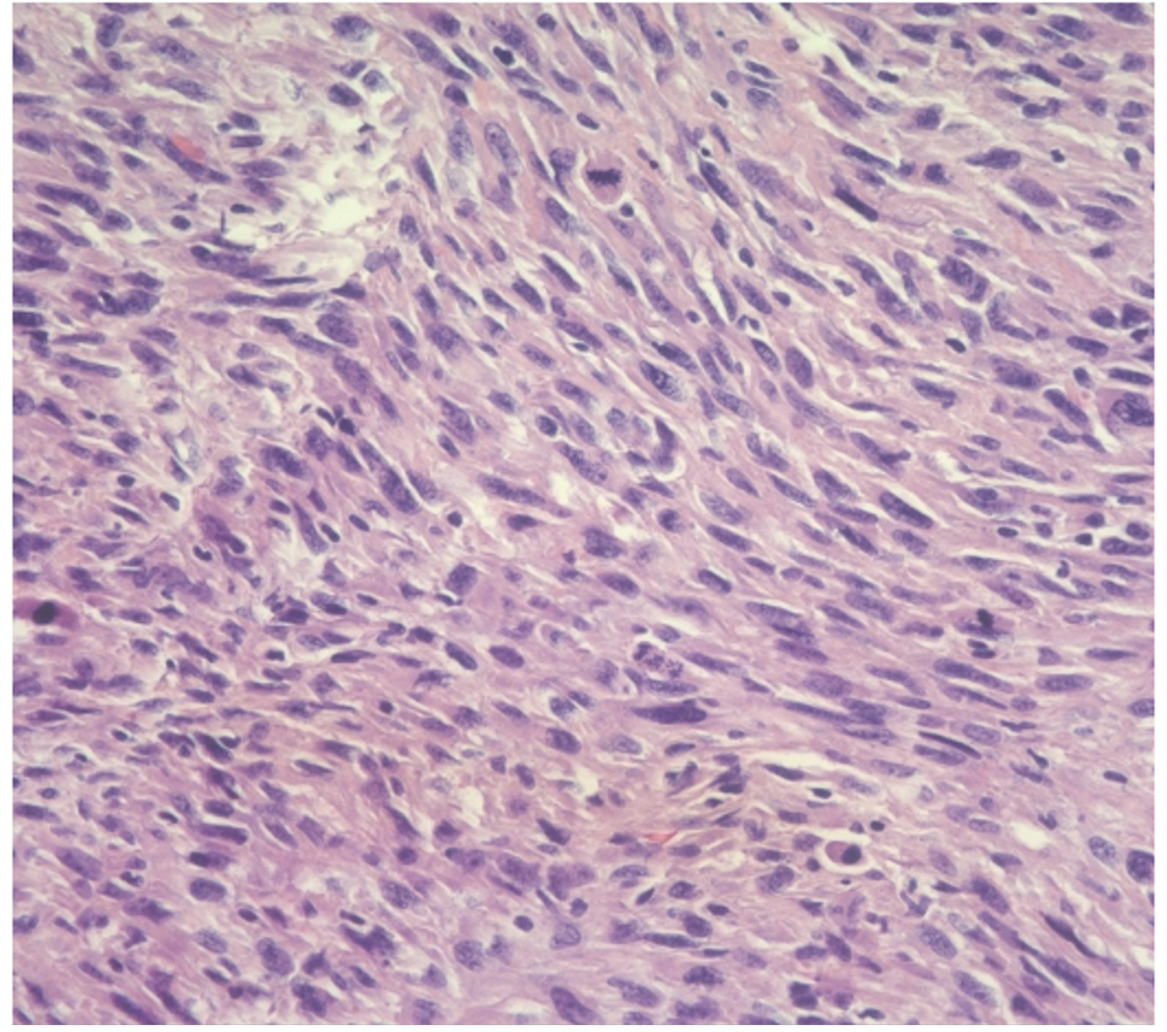
Histopathology Masson's trichrome stain showing neoplastic cells in whorls with pleomorphism and high mitotic activity.

A CT-guided biopsy of the mass was performed and was suspicious for smooth muscle neoplasm. After insertion of a right-sided ureteral stent and improvement of the patient's symptoms, she underwent an exploratory laparotomy, which revealed a mass encasing the right mid-ureter, which was resected and underwent subsequent ureteroureterostomy. A complete surgical excision was performed as per operative report; however, tumor cells were present at the inked resection margin, hence, it was classified as an intralesional excision. Pathologic examination also demonstrated neoplastic cells with atypical elongated nuclei with abundant pleomorphism, heterogeneous chromatin, and atypical mitoses. The tumor was graded according to the French Federation of Cancer Centers Sarcoma Group (FNCLCC) on the basis of tumor differentiation (score 1) [8]. Furthermore, the mitotic rate was 26 per 10 high power field (score 3), and necrosis was up to 30% (score 1). This gives an overall score of 5-6 and a grade of 2 (Figure 2). 

**Figure 3:**
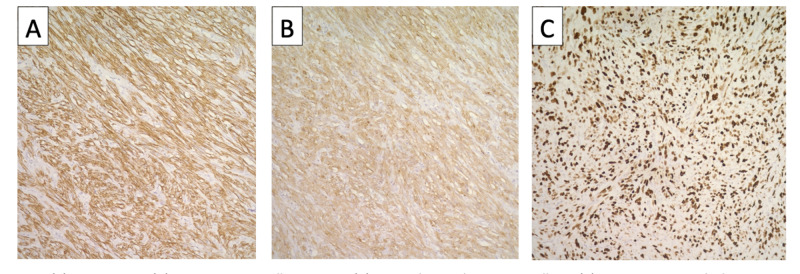
Immunohistochemistry stains (A) Actin stain in yellow brown, (B) smooth muscle actin in yellow, and (C) Ki69 stain in dark showing high mitotic activity nuclei.

IHC staining of the tumor cells was strongly positive for actin, smooth muscle actin (SMA), and vimentin and focally positive for desmin and caldesmon (Figure 3); however, the neuronal marker S100 and the interstitial markers CD117 and CD34 were negative. Ki-67 index was very high at 90%, suggestive of high mitotic activity. This histopathological picture is most compatible with mesenteric LMS.

The patient remained disease-free for one year, until she presented with recurrent nausea, vomiting, diarrhea, and diffuse abdominal pain. A repeat CT scan of the abdomen and pelvis was worrisome for tumor recurrence, as it revealed a 2.0 x 1.8 x 2.7 cm^3^ enhancing mass compressing the right ureter and causing hydronephrosis. Exploratory laparotomy was performed, and a 3 cm retroperitoneal right lower quadrant mass was found and appeared to be arising from the ascending mesocolon with right ureter obstruction. She underwent right hemicolectomy and right nephrectomy and pathology confirmed recurrence. Unfortunately, the patient developed postoperative complications resulting in her death six days postoperatively.

## Review

Mesenteric LMS was first described in 1963 by Kato et al. and is derived from the smooth muscle of mesenteric blood vessels [5], which may explain the frequent hematogenous metastasis. Mesenteric LMS most commonly involves the meso-ileum, but there are rare reported cases involving the transverse, ascending, descending, and sigmoid mesocolon as well as the omentum and gastro-hepatic ligament [1,5,9-11]. While the presentation of LMS is often vague and variable, it most commonly presents with obstructive symptoms such as nausea, vomiting, diarrhea, abdominal distention, and nonspecific abdominal pain. Many patients also have palpable abdominal masses and unintentional weight loss [3,4,9]. Approximately 50% of patients will develop distant metastasis, most commonly to the liver, and lungs [3,9,11], and rarely to adjacent lymph nodes [7]; therefore, routine lymph node dissection is not recommended.

The prognosis of LMS is quite poor as the overall five-year survival rate is at 20% to 30%, which is thought to be due to the large available space in the abdominal cavity, rendering the tumor undetectable until late in the disease course [3,4,9].

In our current literature review, we performed a PubMed database search using the keyword “mesenteric leiomyosarcoma” and since IHC was introduced in 1998 as an essential element to differentiate between GIST and LMS [5,12-15], any reported cases prior to 1998, were excluded for the current study. The largest case review of mesenteric LMS was reported by Kato et al. and included 13 patients between the years 1999 and 2016 [5]. In our search, we included new cases from 2016 to 2020, and a total of 20 articles were initially identified. After reviewing these articles, 14 articles were excluded from this literature review as they either did not originate from the mesentery [13] or were not in English [1], therefore, a total of six new cases of mesenteric LMS, including our case were compiled.

Literature review results

Data were analyzed after all 19 eligible cases were compiled. The median age of presentation for patients with mesenteric LMS was 55 years (range: 13-86), and 68% (n = 13) were females. When mentioned, patients most commonly reported abdominal pain or palpable abdominal mass. Interestingly, one rare case presented with posterior reversible encephalopathy syndrome (PRES) [3]. The most commonly encountered location is the small intestine mesentery (seven cases), followed by the meso-sigmoid (five cases), ascending mesocolon (two cases), descending mesocolon (one case), mesorectum (one case), and 16% (three cases) only stated location as “mesenteric,” without specification (Table 1).

**Table 1:** Demography and treatment Summary for previously published case report demographic and management. ^PRES: posterior reversible encephalopathy syndrome, M: male, F: female, NR: not reported.^

Age/gender	Presentation	Size (mm)	Chemotherapy	Metastasis	Interval between initial diagnosis and metastasis (months)	Recurrence of primary	Interval between initial diagnosis and recurrence (months)	Documented follow-up (months)	Reference #
50 F	Mass, change in bowel habits	140	NR	None	NR	No	NR	6	[1]
82 F	Mass, pain	110	No	Liver	24	No	NR	24	[2]
52 F	Pain, PRES	100	Yes	Liver, lung	0	Yes	5	20	[3]
62 F	Mass	140	Yes	Liver	0	NR	NR	10	[4]
76 F	Mass	140	No	Liver	24	No	NR	40	[5]
45 F	Mass	140	NR	None	NR	No	NR	9	[6]
40 F	Pain, distension	80	Yes	Liver, lung	3	No	NR	39	[9]
65 M	Mass, pain	200	Yes	Liver, local	11	Yes	3	30	[10]
49 F	NR	NR	NR	Liver	29	NR	NR	59	[11]
46 M	NR	NR	NR	Liver, lung	16	NR	NR	26	[11]
41 M	NR	230	NR	Liver	0	NR	NR	15	[12]
86 M	NR	125	NR	None	NR	NR	NR	3	[12]
46 F	Mass	NR	Yes	Liver, local	48	Yes	6	58	[13]
62 F	N.M	78	Yes	Liver, local	0	NR	NR	24	[13]
62 F	Pain	210	Yes	Omentum	0	NR	NR	NR	[14]
13 M	Mass	100	NR	None	NR	NR	NR	3	[16]
33 M	Pain	150	NR	None	NR	NR	NR	NR	[17]
62 F	Fullness	220	NR	None	NR	No	NR	6	[18]
68 F	Pain, vomiting	57	No	None	NR	Yes	12	12	NR

Although the size of the tumor was not always reported, the largest reported tumor was 230 mm [12], with a median size of 139 mm (range: 57-230 mm). Out of the total cases reported, 42% (n = 8) had liver metastasis, 16% (n = 3) had synchronous liver and lung metastasis, 37% (n = 7) had no metastasis, and 5% (n = 1) had metastasis to the omentum. Of those mentioned, five cases had metastasis seen on the initial diagnosis of the primary tumor. The median interval between initial diagnosis and development of metastasis when reported was 13 months (range: 0-48) and 21% (4 cases) showed recurrence of the primary tumor within a median of 6.5 months of initial diagnosis (range: 3-12 months).

Given that LMSs originate from smooth muscle cells, confirming the diagnosis with IHC is necessary for accurate diagnosis [5]. SMA (n = 17) was positive in 100% of the reported cases. When tested, actin (n = 3), caldesmon (n = 4), and vimentin (n = 5) were all positive. Of those that tested for desmin (n = 17), all except one case were positive. Of the cases that tested for S100 (n = 10) and CD 117 (c-KIT) (n = 14), all were negative. As for CD34 (n = 10), all were negative except one that was minimally positive (Table 2).

**Table 2:** IHC review All reviewed LMS cases and IHC stain report. LMS: leiomyosarcoma, IHC: immunohistochemistry, n/a: no answer, MAL: smooth muscle actin, +: positive, -: negative.

References	Actin	SMA	Caldesmon	Vimentin	Desmin	S100	CD34	CD117
Miettinen et al. [12]	n/a	+	n/a	n/a	+	-	-	-
Miettinen et al. [12]	n/a	+	n/a	n/a	+	-	-	-
Fukanaga [4]	+	+	n/a	+	+	-	-	-
Simonovich et al. [2]	+	n/a	n/a	+	+	n/a	-	-
Iwasaki et al. [16]	n/a	n/a	n/a	+	-	-	-	n/a
Koczkowska et al. [13]	n/a	+	n/a	n/a	+	-	-	-
Koczkowska et al. [13]	n/a	+	n/a	n/a	+	n/a	-	-
Mizobe et al. [10]	n/a	+	n/a	n/a	n/a	-	n/a	-
Sidhic et al. [17]	n/a	_+_	n/a	n/a	n/a	n/a	-	-
Hamed et al. [11]	n/a	+	n/a	n/a	+	n/a	n/a	-
Hamed et al. [11]	n/a	+	n/a	n/a	+	n/a	n/a	-
Dasgupta et al. [18]	n/a	+	n/a	n/a	+	n/a	n/a	-
Kato et al. [5]	n/a	+	n/a	n/a	+	-	n/a	-
Varghese et al. [9]	n/a	+	+	n/a	+	n/a	n/a	n/a
Ilias et al. [6]	n/a	+	+	n/a	+	-	+	-
Dalal et al. [1]	n/a	+	n/a	+	+	-	-	-
Schoucair et al. [3]	n/a	+	+	n/a	+	n/a	n/a	n/a
Yoon et al. [14]	n/a	+	n/a	n/a	+	n/a	n/a	n/a
Our reported case	+	+	+	+	+	-	n/a	n/a

 

All patients were managed with surgical resection, apart from two cases where modality of treatment was not reported. The extent of resection was dependent on whether there is an invasion of adjacent structures. Only 37% (7 cases) underwent chemotherapy and of those, six cases underwent palliative/salvage chemotherapy [3,4,9,10,13], and a patient had adjuvant chemotherapy [14]. Of those who underwent salvage chemotherapy, two discontinued therapy due to side effects and subsequently died due to disease progression [13], three patients developed new liver metastasis after chemotherapy [3,4,10], and only one patient showed a response with a decrease in tumor size and subsequently underwent radioablation of the lung and liver masses with a 36-month tumor-free follow up [9]. The response of the patient who underwent adjuvant therapy was not reported [14]. Two out of the seven patients who received salvage chemotherapy did not respond to chemotherapy and subsequently underwent chemoembolization of metastatic lesions [10,13]. These clinical reports put into question the efficacy of chemotherapy in mesenteric LMS but suggest a clinical benefit of chemoembolization. However, this is yet to be established in clinical trials.

Postoperative serial imaging such as a CT chest, abdomen, and pelvis is recommended in LMS. As noticeable in our review, 63% (n = 12) of patients had positive metastasis in a median of 13-months and 21% (n = 4) showed recurrence of primary tumor within a median of 6.5 months (Table 1). Therefore, in patients who underwent complete tumor resection, strategic surveillance is recommended every three to six months for two to three years, and then annually [7]. Recommendations in those with positive margins include a physical examination with abdominal and pelvic imaging every three to six months for two to three years, then six months for the next two years, and then annually. Moreover, due to the high rate of lung metastasis, CT of the chest is also indicated annually. 

The median follow-up time for the cases presented (n = 17) was 22.6 months (range: 3-59), and consistent with the findings of Kato et al. [5], no patient was reported to survive 59 months (Table 1) [11].

## Conclusions

Mesenteric LMS are rare tumors, with aggressive behavior as manifested by their high metastatic rate. The clinical course appears to be variable based on location and histologic characteristics. Differentiating LMS from GISTs histologically can be challenging, given the diversity in morphology. Perhaps, the best way of separating the two entities is by IHC. As LMS are derived from smooth muscle cells, they stain positively for smooth muscle markers and negatively for intestinal markers. Conversely, GISTs arise from the interstitial cells of Cajal and stain positively with C-kit, DOG1, and CD34.

In this report, we review the common clinical and diagnostic characteristics of mesenteric LMS and highlight the limitation of our knowledge in this rare entity. Therefore, further research is needed to optimize management and improve the prognosis of LMS.
